# Prevention of SARS-CoV-2 transmission from international arrivals: Xiaotangshan Designated Hospital, China

**DOI:** 10.2471/BLT.20.265918

**Published:** 2021-03-02

**Authors:** Zujin Luo, Yi Zhang, Yue Zheng, C Raina MacIntyre, Ying Liang, Quanyi Wang, Yingmin Ma

**Affiliations:** a Department of Respiratory and Critical Care Medicine, Beijing Chao-Yang Hospital, Capital Medical University, No. 5 Jingyuan Road, Shijingshan District, Beijing 100043, China.; b Institute for Infectious Disease and Endemic Disease Control, Beijing Center for Disease Prevention and Control, Beijing, China.; c Department of Surgical Intensive Care Unit, Beijing Chao-Yang Hospital, Beijing, China.; d Kirby Institute, University of New South Wales, Sydney, Australia.; e Department of Internal Medicine, Beijing Xiaotangshan Hospital, Beijing, China.

## Abstract

A surge in the number of international arrivals awaiting coronavirus disease 2019 (COVID-19) screening overwhelmed health-care workers and depleted medical resources in designated hospitals in Beijing, China in March 2020. The People’s Government of Beijing Municipality therefore issued a policy which required the mandatory transfer of all asymptomatic passengers arriving from a foreign country to designated quarantine hotels, and the transfer of passengers with fever or respiratory symptoms to designated hospitals. Xiaotangshan Designated Hospital, a severe acute respiratory syndrome hospital in 2003, was rapidly renovated and put into operation with the main tasks of screening and isolating symptomatic international arrivals at Beijing Capital International Airport, providing basic medical care for mild to moderate COVID-19-positive cases, and rapidly referring severe to critical COVID-19-positive cases to higher-level hospitals. During the month-long period of its operation, 2171 passengers were screened and 53 were confirmed as having COVID-19 (six severe to critical). We describe how the use of Xiaotangshan Designated Hospital in this way enabled the efficient grouping and assessment of passengers arriving from a foreign country, the provision of optimal patient care without compromising public safety and the prioritization of critically ill patients requiring life-saving treatment. The designated hospital is a successful example of the World Health Organization’s recommendation to renovate existing medical infrastructures to improve the COVID-19 response capacity. The flexible design of Xiaotangshan Designated Hospital means that it can be repurposed and reopened at any time to respond to the changing pandemic conditions.

## Résumé

En mars 2020, la brusque hausse du nombre d'arrivées internationales en attente de dépistage de la maladie à coronavirus 2019 (COVID-19) a submergé les professionnels de la santé et épuisé les ressources médicales dans les hôpitaux de référence à Beijing, en Chine. Le gouvernement populaire de la municipalité de Beijing a réagi en ordonnant que tous les passagers asymptomatiques en provenance d'un pays étranger soient transférés vers des hôtels reconvertis en centres de quarantaine, et que ceux manifestant de la fièvre ou des symptômes respiratoires soient envoyés dans des hôpitaux de référence. L'hôpital de référence Xiaotangshan, construit en 2003 pour lutter contre le syndrome respiratoire aigu sévère, a rapidement été rénové et mis en service. Ses tâches principales: dépister et isoler les passagers internationaux symptomatiques débarquant au Beijing Capital International Airport, prodiguer les soins médicaux de base aux cas positifs de COVID-19 souffrant d'une forme légère à modérée, et adresser dès que possible les cas positifs de COVID-19 dans un état grave ou critique aux hôpitaux spécialisés. En l'espace d'un mois, 2171 passagers ont été testés et 53 se sont révélés positifs à la COVID-19 (6 étant dans un état grave ou critique). Nous décrivons la façon dont l'hôpital de référence Xiaotangshan a ainsi permis de regrouper et d'évaluer efficacement les arrivées en provenance de l'étranger, d'offrir une prise en charge optimale des patients sans compromettre la sécurité publique, et d'établir des priorités afin que les malades gravement atteints puissent bénéficier d'un traitement dans les plus brefs délais. Cet hôpital de référence est un exemple réussi de la mise en œuvre de la recommandation formulée par l'Organisation mondiale de la Santé: rénover les infrastructures médicales existantes afin d'améliorer les capacités de lutte contre la COVID-19. Grâce à sa conception flexible, l'hôpital Xiaotangshan peut être réutilisé et rouvert à n'importe quel moment pour réagir à un contexte pandémique en perpétuelle évolution.

## Resumen

Un aumento del número de llegadas de vuelos internacionales en espera de la detección del coronavirus 2019 (COVID-19) sobrecargó al personal sanitario y agotó los recursos médicos en los hospitales designados de Pekín (China) en marzo de 2020. Por lo tanto, la policía del Gobierno Popular del municipio de Pekín se tuvo que hacer cargo del traslado obligatorio de todos los pasajeros asintomáticos que llegaran de un país extranjero a los hoteles de cuarentena designados, y el traslado de los pasajeros con fiebre o síntomas respiratorios a los hospitales designados. El hospital designado de Xiaotangshan, un hospital especializado en el síndrome respiratorio agudo severo en 2003, se rehabilitó rápidamente y se puso en funcionamiento con las tareas principales de examinar y aislar a los sintomáticos que llegaban al Aeropuerto Internacional de Pekín, proporcionando atención médica básica a los casos positivos de COVID-19 de leves a moderados, y derivando rápidamente los casos positivos de COVID-19 de graves a críticos a hospitales de nivel superior. Durante el mes que duró su funcionamiento, se examinó a 2.171 pasajeros y se confirmó que 53 tenían la COVID-19 (6 de ellos con intensidad de grave a crítica). Describimos cómo el uso del hospital designado de Xiaotangshan permitió agrupar y evaluar eficazmente a los pasajeros que llegaban de un país extranjero, prestar una atención óptima a los pacientes sin comprometer la seguridad pública y priorizar a los pacientes en estado crítico que requerían tratamiento para salvar su vida. El hospital designado es un ejemplo de éxito de la recomendación de la Organización Mundial de la Salud de renovar las infraestructuras médicas existentes para mejorar la capacidad de respuesta ante la COVID-19. El diseño flexible del hospital designado de Xiaotangshan significa que puede utilizarse y volver a habilitarse en cualquier momento para responder a las condiciones cambiantes de la pandemia

## ملخص

أدت الزيادة في عدد الرحلات الدولية القادمة التي بانتظار الفحص الخاص بمرض كورونا فيروس 2019 (كوفيد 19) إلى إرهاق أخصائيي الرعاية الصحية واستنفاد الموارد الطبية في المستشفيات المخصصة في مدينة بكين في الصين في شهر مارس/آذار 2020. ولهذا اتجهت سياسة حكومة الشعب في بلدية بكين إلى النقل الإلزامي لكل الركاب الذين لا تظهر عليهم أعراض من القادمين من بلد أجنبي إلى فنادق مخصصة للعزل ونقل الركاب المصابين بحمى أو أعراض في الجهاز التنفسي إلى المستشفيات المخصصة. شهدت مستشفى زياوتانغشان المخصصة ـ وهي مستشفى لمتلازمة الجهاز التنفسي الحادة الشديدة تم إنشاؤها في عام 2003 ـ تجديدًا سريعًا وتم إدخالها للخدمة للقيام بمهام رئيسية تتمثل في فحص الرحلات الدولية القادمة التي تظهر فيها أعراض في مطار العاصمة الدولي في بكين مع تقديم الرعاية الطبية الأساسية للحالات الخفيفة إلى المتوسطة من الإصابة بكوفيد 19، وإحالة الحالات الحادة إلى الحرجة من الإصابة بكوفيد 19 سريعًا إلى مستشفيات أعلى في المستوى. على مدار فترة تشغيلها التي امتدت شهرًا، تم فحص 2171 راكبًا وتم تأكيد إصابة 53 منهم بكوفيد 19 (6 منهم بحالات حادة إلى حرجة). نقدم وصفًا لطريقة استخدام مستشفى زياوتانغشان المخصصة بهذه الطريقة مما أدى إلى التمكن من تصنيف الركاب القادمين من بلد أجنبي وتقييمهم بكفاءة، وتقديم الرعاية الأمثل للمريض من دون التأثير على السلامة العامة وتحديد أولوية المرضى ذوي الحالات الحرجة التي تتطلب علاجًا ينقذ حياتهم. تمثل المستشفى المخصصة مثالاً ناجحًا لتوصية منظمة الصحة العالمية لتجديد البنى التحتية الطبية القائمة من أجل تحسين القدرة على الاستجابة لكوفيد 19. يقدم التصميم المرن لمستشفى زياوتانغشان المخصصة إمكانية تغيير الغرض منها وإعادة افتتاحها في أي وقت للاستجابة للحالات الوبائية المتغيرة.

## 摘要

在 2020 年 3 月，随着等待接受新型冠状病毒肺炎 (COVID-19) 筛查的境外输入人口激增，中国北京定点医院的医护人员感到不堪重负，且医疗资源几近枯竭。鉴于此，北京市人民政府制定了政策，要求将所有无症状的境外输入旅客转移至定点隔离酒店，并将有发烧或呼吸道症状的旅客转移至定点医院。小汤山定点医院是 2003 年治疗严重急性呼吸综合征（非典）的定点医院，经快速翻新后，该医院投入运营，其主要任务是筛查和隔离北京首都国际机场的有症状境外输入人员，为轻度至中度新型冠状病毒肺炎阳性病例提供基础医疗服务，并将重度至危重型新型冠状病毒肺炎阳性病例迅速转诊至更高级别的医院。在其运营的一个月时间内，共对 2171 名旅客进行了筛查，并有 53 名旅客被确诊患有新型冠状病毒肺炎（6 例重度至危重病例）。我们描述了如何利用小汤山定点医院对境外输入旅客有效地分组和评估，在不损害公共安全的前提下提供最佳的患者护理，以及优先处理需要挽救生命的危重患者。该定点医院在翻新现有医疗基础设施以提高新型冠状病毒肺炎响应能力方面取得了全面成功，是世界卫生组织推荐学习的示范医院。小汤山定点医院的灵活设计意味着可以随时调整用途并重新开放，以应对不断变化的疫情状况。****

## Резюме

В связи с быстрым ростом числа лиц, прибывающих из-за границы и ожидающих скринингового обследования коронавирусной болезни 2019 г. (COVID-19), медицинские работники оказались перегружены, а медицинские ресурсы истощены в специализированных больницах в Пекине, Китай, в марте 2020 года. Таким образом, политика Народного правительства города Пекина заключалась в обязательном переводе всех бессимптомных пассажиров, прибывающих из другой страны, в специализированные карантинные отели, а также в переводе пассажиров с температурой или респираторными симптомами в специализированные больницы. Специализированная больница Сяотаншань, использовавшаяся для пациентов с тяжелым острым респираторным синдромом в 2003 году, была быстро отремонтирована и сдана в эксплуатацию со следующими основными задачами: скрининговое обследование и изоляция пассажиров с симптомами, прибывающих из-за границы в международный аэропорт Пекина Шоуду, оказание базовой медико-санитарной помощи положительным по COVID-19 пациентам с легкой и умеренной формами заболевания, а также быстрое направление положительных по COVID-19 пациентов в тяжелой и критической форме в больницы более высокого уровня. За месяц работы обследование прошел 2171 пассажир, у 53 пассажиров подтверждено наличие COVID-19 (у 6 пассажиров в форме от тяжелой до критической). Мы описываем, каким образом использование специализированной больницы Сяотаншань позволило эффективно группировать и оценивать пассажиров, прибывающих из другой страны, обеспечивать оптимальную помощь пациентам без ущерба для общественной безопасности и уделять приоритетное внимание тяжелобольным пациентам, нуждающимся в жизненно необходимых процедурах лечения. Специализированная больница является успешным примером рекомендации Всемирной организации здравоохранения по обновлению существующей медицинской инфраструктуры для улучшения потенциала реагирования на COVID-19. Гибкая структура специализированной больницы в Сяотаншане означает возможность ее перепрофилирования и повторного открытия в любое время для реагирования на изменяющиеся условия пандемии.

## Introduction

**Fig. 1:**
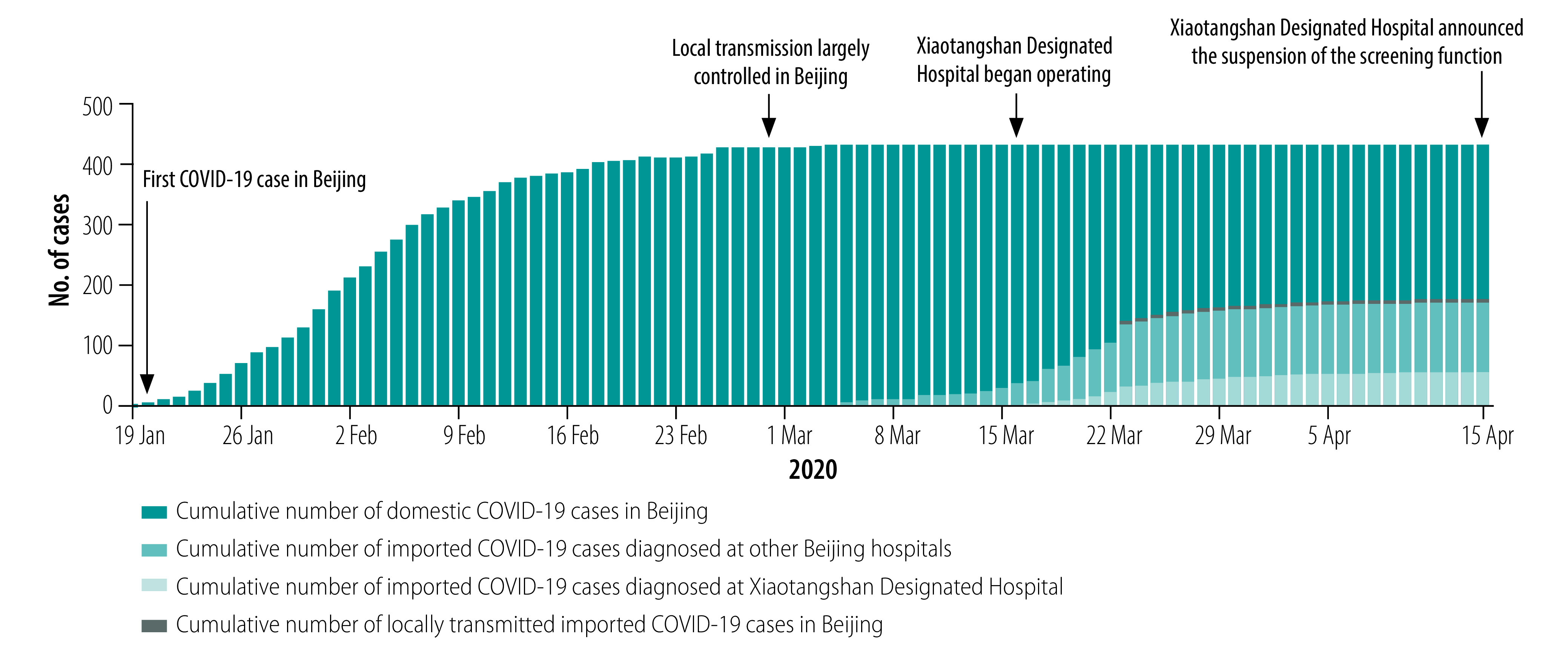
Cumulative number of COVID-19 domestic cases, and of cases diagnosed among international arrivals at Xiaotangshan Designated Hospital and other hospitals, January–April 2020, Beijing, China

The World Health Organization (WHO) declared the coronavirus disease (COVID-19) outbreak as a public health emergency of international concern on 30 January 2020.^1^ At that date, 7818 cases had been confirmed globally.^2^ In Beijing, local transmission of severe acute respiratory syndrome coronavirus 2 (SARS-CoV-2) was contained by the end of February 2020, after 428 cases had been reported (Fig. 1). However, a combination of the alarming level of transmission in foreign countries and the fact that Beijing is a major hub of international aviation meant that the city faced the potential reintroduction of the virus.^3^ Screening and isolating cases arriving from a foreign country therefore became the top priority. 

COVID-19: coronavirus disease.

Data source: Beijing Center for Disease Prevention and Control; Beijing Xiaotangshan Designated Hospital.

During March 2020, around 100 000 passengers (4000 with symptoms) arrived at Beijing Capital International Airport and required quarantine or screening, placing a huge burden on established COVID-19 designated hospitals. All screening was initially performed at Beijing Ditan Hospital, a traditional infectious disease hospital and a higher-level hospital for treating severely ill COVID-19 patients.^4^ However, the surge in the number of arrivals requiring to be screened led to the diversion of medical resources originally allocated for the treatment of existing patients with severe COVID-19; health-care workers became overwhelmed and the supplies of medical equipment, such as nucleic acid detection tests and computed tomography scanning, were rapidly depleted.^4^

Beijing was in urgent need of a new dedicated facility to increase its capacity to screen and isolate travellers arriving from a foreign country, and to optimize the allocation of medical resources. Xiaotangshan Hospital, located on the outskirts of Beijing adjacent to Beijing Capital International Airport and Beijing Ditan Hospital, was a severe acute respiratory syndrome (SARS) designated hospital in 2003.^5^ The first hospital rapidly built in China to tackle an outbreak of a single infectious disease, it represented a major contribution to China’s response to the public health emergency. After the outbreak of COVID-19, the original Xiaotangshan Hospital model was replicated in Wuhan when the city rapidly built a number of designated hospitals (such as Huoshenshan and Leishenshan) to ease the shortage of beds for COVID-19 patients.^6^^,^^7^ As part of the planning for a possible surge in COVID-19 cases, Xiaotangshan Hospital was expanded, renovated and repurposed, and renamed Xiaotangshan Designated Hospital by the People’s Government of Beijing Municipality in January 2020.^8^^–^^11^ The designated hospital was formally put into operation on 16 March 2020 to screen and isolate arrivals at Beijing Capital International Airport with fever or respiratory symptoms; provide basic medical care for mild to moderate confirmed COVID-19 cases; and rapidly refer severe to critical confirmed COVID-19 cases to higher-level hospitals.

On 11 March 2020, the People’s Government of Beijing Municipality issued a policy which required the mandatory transfer of all asymptomatic travellers arriving from a foreign country to designated quarantine hotels, and the transfer of passengers with fever or respiratory symptoms to designated hospitals. Although an important alternative to hospital isolation,^12^ voluntary self-isolation of travellers is unlikely to be fully effective because of a general lack of adherence to the required living conditions; family members are put at risk and self-isolators can experience psychological stress.^12^^,^^13^ Further justification for quarantining at designated sites is that unnecessary delays are avoided for any required medical care or even rapid referral to a hospital.^13^

Xiaotangshan Designated Hospital, together with Beijing Ditan Hospital, designated quarantine hotels and dedicated transfer vehicles, comprised the primary medical system in the prevention of virus transmission from international arrivals at Beijing Capital International Airport. We describe the structure and design of Xiaotangshan Designated Hospital, and the resources available to it. We also explain the function and operation of the designated hospital, and discuss its effectiveness and value in responding to the pandemic and preventing the transmission of the virus from overseas travellers.

## Structure and resources

**Fig. 2:**
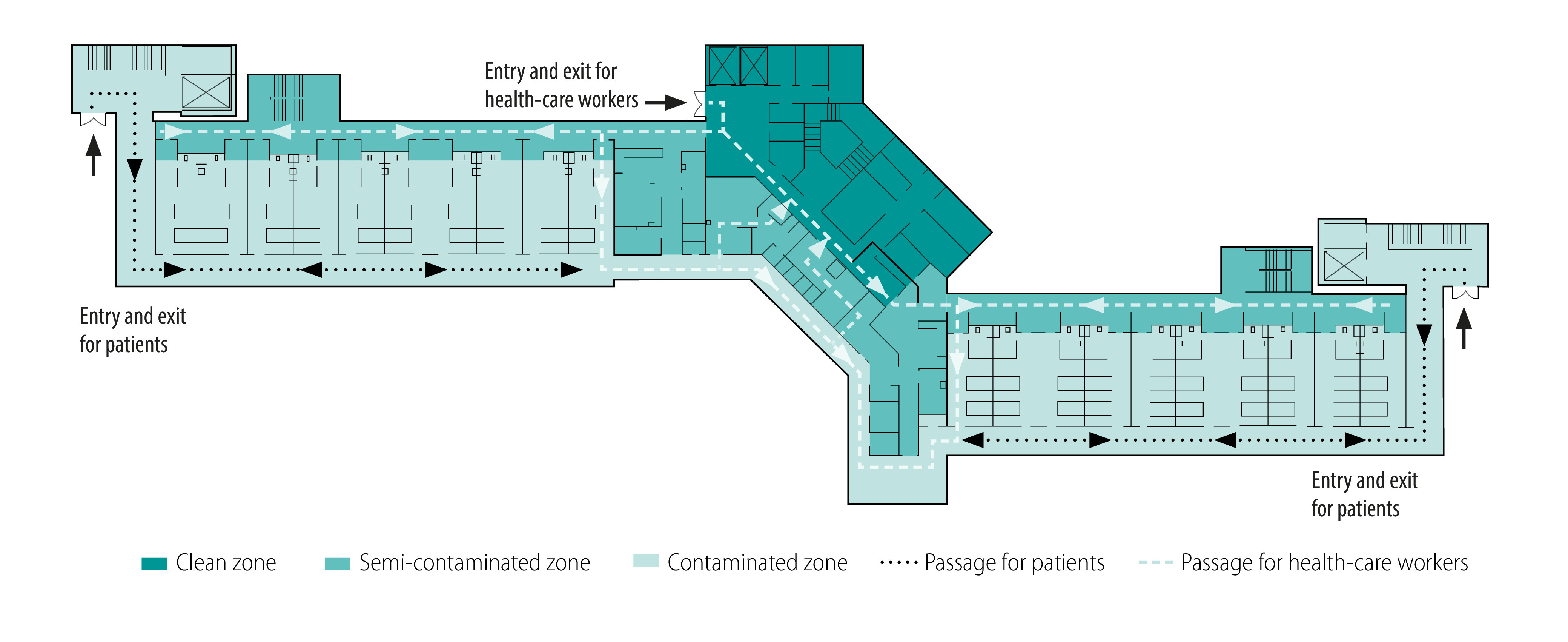
Floor plan of a subunit of Xiaotangshan Designated Hospital for isolation of symptomatic travellers arriving from a foreign country while screening for COVID-19, March–April 2020, Beijing, China

Xiaotangshan Designated Hospital consisted of two separate medical areas: a screening area with 750 single-bed wards, and a treatment area with nearly 150 beds. Both areas included three separate contamination-level zones: a contaminated zone where patients resided; a semi-contaminated zone where health-care workers removed personal protective equipment; and a clean zone where health-care workers received supplies, performed clerical work and rested (Fig. 2). Both areas also included two separate corridors linking the infectious disease isolation wards:^13^^–^^16^ one corridor for use by isolating travellers and another for health-care workers (Fig. 2). 

COVID-19: coronavirus disease.

Xiaotangshan Designated Hospital was well equipped with oxygen therapy devices, mechanical ventilators, and nucleic acid detection and computed tomography scanning equipment. To ensure efficient operation of the hospital and to maximize its screening capacity for COVID-19, it was crucial to have sufficient human resources. Beijing authorities mobilized health-care workers from 22 hospitals across the city to support Xiaotangshan Designated Hospital.^17^ Health-care workers responded positively regardless of their circumstances;^18^ many even volunteered their services for the designated hospital, despite the known risk of infection. All health-care workers received rigorous pre-employment professional training that included: identifying the symptoms of COVID-19; understanding the guidelines regarding COVID-19 diagnosis and treatment; practising the standard process for wearing and removing personal protective equipment; and being aware of the correct corridors to use to access the isolation wards.

The People’s Government of Beijing Municipality convened a leadership group for COVID-19 in Xiaotangshan Designated Hospital. This group, led by the deputy director of the Beijing Municipal Health Commission, was created to manage the hospital’s daily operations. The local government coordinated with various departments to ensure that food and medical supplies were available for both health-care workers and isolating travellers, and that basic facilities such as water, electricity and security were available.^17^ To ensure that the public understood and supported the mission of Xiaotangshan Designated Hospital, the hospital management team held several press conferences and arranged multiple interviews with officials and health-care workers to provide information about the hospital.

## Function and operation

### Screening

Travellers arriving at Beijing Capital International Airport with fever or respiratory symptoms were transferred to Xiaotangshan Designated Hospital, and tested for SARS-CoV-2 nucleic acid in combined nasopharyngeal and oropharyngeal swabs and serum antibodies; passengers also received chest computed tomography scans and routine blood tests. COVID-19 was confirmed if passengers tested positive for SARS-CoV-2 nucleic acid or their serum immunoglobulin M and immunoglobulin G were both positive.^19^ During screening, which took an average of 30 hours (from 11 hours minimum to over 5 days), all passengers were strictly subjected to single-room quarantine to avoid nosocomial infection of passengers whose symptoms were unrelated to SARS-CoV-2. As well as food and accommodation, within the screening area passengers could also access basic medical care, a disease consultant and emotional support.

COVID-19 was ruled out if both the nucleic acid test and any serum antibodies were negative, as well as no obvious abnormalities on the chest scan and in the blood test.^19^COVID-19-negative passengers were transferred to designated quarantine hotels by dedicated transfer vehicles for a 14-day observation period. If passengers were asymptomatic and had a negative nucleic acid test on day 14, they could return to their homes by dedicated transfer vehicle. To prevent the transmission of COVID-19 with a possible incubation period of more than 14 days, self-isolation was recommended for an additional 14 days.^20^ Anyone who developed a fever or respiratory symptoms during hotel quarantine was returned to the screening area of Xiaotangshan Designated Hospital for COVID-19 testing.

### Mild and moderate cases

If mild (with symptoms including cough, sore throat, fatigue, myalgia or headache, and no signs of pneumonia on chest scan) or moderate (with symptoms also including fever and dyspnoea and pneumonia evident from the chest scan) COVID-19 was diagnosed, patients were transferred from the screening area to the treatment area of the designated hospital via a dedicated in-hospital passage for isolation and treatment.^19^ Patients received basic medical care (including antiviral, antibiotic, antipyretic and traditional Chinese medicine) as well as intravenous fluids, conventional and high-flow oxygen supplementation and respiratory rehabilitation. Health-care workers measured the body temperature, heart rate, blood pressure, respiratory rate and oxygen saturation of patients more than four times per day to monitor the progression of the disease. Basic life support (including cardiopulmonary resuscitation, rapid defibrillation, endotracheal intubation and subsequent invasive ventilation) and close electrocardiographic monitoring were provided in two temporary single-bed intensive care units for rapidly deteriorating patients. In addition to basic food and accommodation, patients could also access the Internet as well as any required emotional support. For professional guidance, the clinical conditions of all patients were reported to a higher-level specialist group in Beijing during an online consultation every day.

### Discharge conditions

Patients in the treatment area were discharged if they met all of the following criteria:^19^ (i) no fever for > 3 days; (ii) significant improvement in respiratory symptoms; (iii) obvious absorption of inflammation on chest scan; and (iv) two consecutive negative nucleic acid tests for SARS-CoV-2 with a sampling interval of more than 24 hours. Discharged patients were then transferred to a designated quarantine hotel for a 14-day period of medical observation. Self-isolation was then recommended for an additional 14 days after leaving the quarantine hotel.

### Severe and critical cases

Whether during examination in the screening area or the treatment area, patients were quickly referred to a higher-level designated hospital, by dedicated transfer vehicle via a pre-established referral pathway, for intensive care if they met any of the following criteria:^19^ (i) a respiratory rate of ≥ 30/min; (ii) an oxygen saturation as measured by pulse oximetry of ≤ 93%; (iii) an arterial oxygen tension of inspired oxygen of ≤ 300 mmHg; (iv) the expansion of a pulmonary lesion in the chest scan by > 50% within 24–48 hours; or (v) the exacerbation of chronic obstructive pulmonary disease, hypertension, diabetes, coronary heart disease or any other comorbidity requiring advanced medical care.

## Effectiveness and value

As a result of the diversion of international flights from Beijing to other ports of entry in China to spread the burden of screening arrivals over many designated hospitals, COVID-19 screening at Xiaotangshan Designated Hospital was gradually reduced and finally suspended on 15 April 2020.^21^ Xiaotangshan Designated Hospital admitted a total of 2171 passengers for screening during its month-long period of operation (408 passengers in a single day at its peak on 21 March 2020). Because an average of 30 hours is needed to confirm or exclude a diagnosis of COVID-19, a maximum of 685 passengers were temporarily hospitalized in the screening area on 21 March 2020; however, the full capacity of 750 single-bed isolation wards was not reached at any point. Of the 2171 symptomatic passengers who were screened, 2118 tested negative and 53 were confirmed to have COVID-19. Of the 53 confirmed cases, six were referred to a higher-level hospital for intensive care; the remaining 47 (88.7%) mild to moderate COVID-19-positive cases were discharged from the designated hospital by 28 April 2020.^22^^,^^23^

In total, 872 health-care workers staffed the designated hospital, including 102 physicians (specialists in respiratory medicine, infectious disease, critical care medicine, paediatrics or traditional Chinese medicine), 728 nurses and 42 technicians. No health-care workers became infected with COVID-19. 

Xiaotangshan Designated Hospital enabled the efficient grouping and assessing of passengers arriving from a foreign country, as well as the provision of optimal care for all patients without compromising public safety. The designated hospital provided appropriate medical care and isolation for mild to moderate cases, avoiding the risk of transmission associated with a lack of adherence to self-isolation.^12^^,^^24^ Further, by screening travellers arriving from a foreign country for COVID-19 and treating patients with mild to moderate SARS-CoV-2 infection (which accounted for almost 90% of all COVID-19-positive arrivals),^7^ the burden on higher-level designated hospitals was reduced, ensuring that critically ill patients who required life-saving treatment could be prioritized.^4^ In other words, the Xiaotangshan Designated Hospital enabled the allocation of Beijing’s medical resources in terms of its response to COVID-19 to be fully optimized.

During the operation of Xiaotangshan Designated Hospital, only one case (whose incubation period was > 14 days) was missed. This patient transmitted the virus to three family members after returning home.^25^ The infrequency of such events highlights the importance of timely screening, isolation and treatment of imported COVID-19 cases.

WHO recommended in March 2020 that new treatment areas or separate hospitals be established to enhance the response to COVID-19, and that more patient care areas in the health system be repurposed for treating COVID-19, especially severe or critical cases.^26^ The opening of Xiaotangshan Designated Hospital was consistent with these recommendations. Other countries with international ports could consider this model of COVID-19 screening, triage, isolation and treatment as part of their public health response to imported cases. 

Xiaotangshan Designated Hospital accomplished its mission of responding to the threat of the reintroduction of the virus from a foreign country. In the future, the hospital could be used to respond to other public health emergencies, such as clustered food poisoning or natural disasters. Alternatively, the hospital could be used as a rehabilitation hospital or physical examination centre. In the meantime, the pandemic is not yet over and COVID-19 is still spreading globally; however, the flexible design of Xiaotangshan Designated Hospital means that it can be repurposed according to current conditions and reopened at any time. 
